# Can a proposed double branch multimodality-contribution-aware TripNet improve the prediction performance of the microvascular invasion of hepatocellular carcinoma based on small samples?

**DOI:** 10.3389/fonc.2022.1035775

**Published:** 2022-10-24

**Authors:** Yuhui Deng, Xibin Jia, Gaoyuan Yu, Jian Hou, Hui Xu, Ahong Ren, Zhenchang Wang, Dawei Yang, Zhenghan Yang

**Affiliations:** ^1^ Department of Radiology, Beijing Friendship Hospital, Capital Medical University, Beijing, China; ^2^ Medical Imaging Division, Heilongjiang Provincial Hospital, Harbin Institute of Technology, Harbin, China; ^3^ Faculty of Information Technology, Beijing University of Technology, Beijing, China; ^4^ Department of Radiology, The People’s Hospital of Jimo.Qingdao, Qingdao, China

**Keywords:** hepatocellular carcinoma, CT, deep learning, MRI, microvascular invasion

## Abstract

**Objectives:**

To evaluate the potential improvement of prediction performance of a proposed double branch multimodality-contribution-aware TripNet (MCAT) in microvascular invasion (MVI) of hepatocellular carcinoma (HCC) based on a small sample.

**Methods:**

In this retrospective study, 121 HCCs from 103 consecutive patients were included, with 44 MVI positive and 77 MVI negative, respectively. A MCAT model aiming to improve the accuracy of deep neural network and alleviate the negative effect of small sample size was proposed and the improvement of MCAT model was verified among comparisons between MCAT and other used deep neural networks including 2DCNN (two-dimentional convolutional neural network), ResNet (residual neural network) and SENet (squeeze-and-excitation network), respectively.

**Results:**

Through validation, the AUC value of MCAT is significantly higher than 2DCNN based on CT, MRI, and both imaging (P < 0.001 for all). The AUC value of model with single branch pretraining based on small samples is significantly higher than model with end-to-end training in CT branch and double branch (0.62 vs 0.69, p=0.016, 0.65 vs 0.83, p=0.010, respectively). The AUC value of the double branch MCAT based on both CT and MRI imaging (0.83) was significantly higher than that of the CT branch MCAT (0.69) and MRI branch MCAT (0.73) (P < 0.001, P = 0.03, respectively), which was also significantly higher than common-used ReNet (0.67) and SENet (0.70) model (P < 0.001, P = 0.005, respectively).

**Conclusion:**

A proposed Double branch MCAT model based on a small sample can improve the effectiveness in comparison to other deep neural networks or single branch MCAT model, providing a potential solution for scenarios such as small-sample deep learning and fusion of multiple imaging modalities.

## Introduction

As one of the most common primary liver malignancies, hepatocellular carcinoma (HCC) is the third leading cause of tumour-related deaths worldwide (1, 2). The optimal treatment choices for HCC like surgical resection and transplantation have been consistently improved in recent decades. However, due to high recurrence rates, early recurrence and long-term prognoses remain unsatisfactory (3). Among several factors, such as histological grade and tumour size, previous studies (4, 5) have confirmed that microvascular invasion (MVI) is a vital factor for early recurrence and poor long-term prognosis in HCC patients treated by resection or transplantation (6, 7). However, the preoperative evaluation of MVI is difficult, which warrants a noninvasive, highly accurate tool for evaluating the presence/absence of MVI in HCC patients when making treatment decisions preoperatively.

Previous studies (8–11) have attempted to predict the presence of MVI based either on computed tomography (CT) or MRI imaging features alone. Although promising results were presented, several limitations still existed to negatively affect the diagnostic performance and clinical applicability to some degree. For example, considerable interobserver variability were found in the assessment of MVI in HCC using MRI (12), even for more experienced radiologists due to inevitable subjective bias in the process of personal imaging analysis. In addition, few study have attempted to predict MVI in HCC based on multi-phase CT and multi-sequence MRI techniques together. Previous studies have seperately found that CT-based features like tumor margin (13) or MRI-based features like ADC value (8) and peritumor hypointensity in the hepatobiliary phase (HBP) (14) had moderate to high correlation with the presence of MVI in HCC. As the presence of MVI in HCC co-exists with diverse radiologic features simultaneously, it is necessary to investigate whether or not the noninvasive prediction accuracy of MVI in HCC could be improved based on a combination of CT and MRI imaging features rather than on a single imaging modality alone.

With the rapid development of machine learning, recent studies have attempted to explore the potential of machine learning including deep learning (15, 16) and radiomics (17, 18) in prediction of MVI in HCC. Based on various deep learning models, several studies found that the deep learning models could achieve a moderate diagnostic accuracy in a range from 0.66 to 0.76 on CT or MRI imaging alone (19–21). However, the further improvement of prediction accuracy of deep learning model for MVI in HCC is hampered by several factors, in which the limited well-annotated medical imaging data is a major one. It is well-known that at least10 thousand data are required for deep learning model to achieve a relatively optimal training and verification results. Nevertheless, even the largest sample size of 750 cases in a published multi-center study (19) is still far from the needs for deep learning. Moreover, the substantial increase of well-annotated medical imaging data to the requirement of deep learning is deemed a genuine hardship considering the far from enough qualified imaging data and high time-consuming for well annotation. In addition, common deep learning models give the same weight to each sequence channel in the diagnosis process of medical problems, rather than assigning different weights to different sequences according to their importance like the diagnostic logic applied by radiologists. Theoretically, diagnostic experiences from radiologists can improve the construction of deep neural networks and alleviate the problem caused by insufficient training samples to a large extent.

To solve the problems mentioned above, we propose a new double-branch multimodality-contribution-aware TripNet (MCAT) model. In this model, the data augmentation and metric learning techniques were applied to overcome the negative effect of small sample size, and the incorporated radiologists’ diagnostic experience with modal (sequence) attention schemes was used to improve the accuracy of deep neural network and alleviate the effect caused by insufficient training samples. It is hypothesized that the MCAT model could improve the prediction accuracy of MVI in HCC compared to other commonly-used deep neural networks on small samples.

Therefore, this study aimed to investigate the possible improvement of prediction performance of MCAT model in MVI of HCC compared to other deep neural networks, based on a multi-modality CT and MRI data with small samples.

## Materials and methods

### Patients

**Figure 1:**
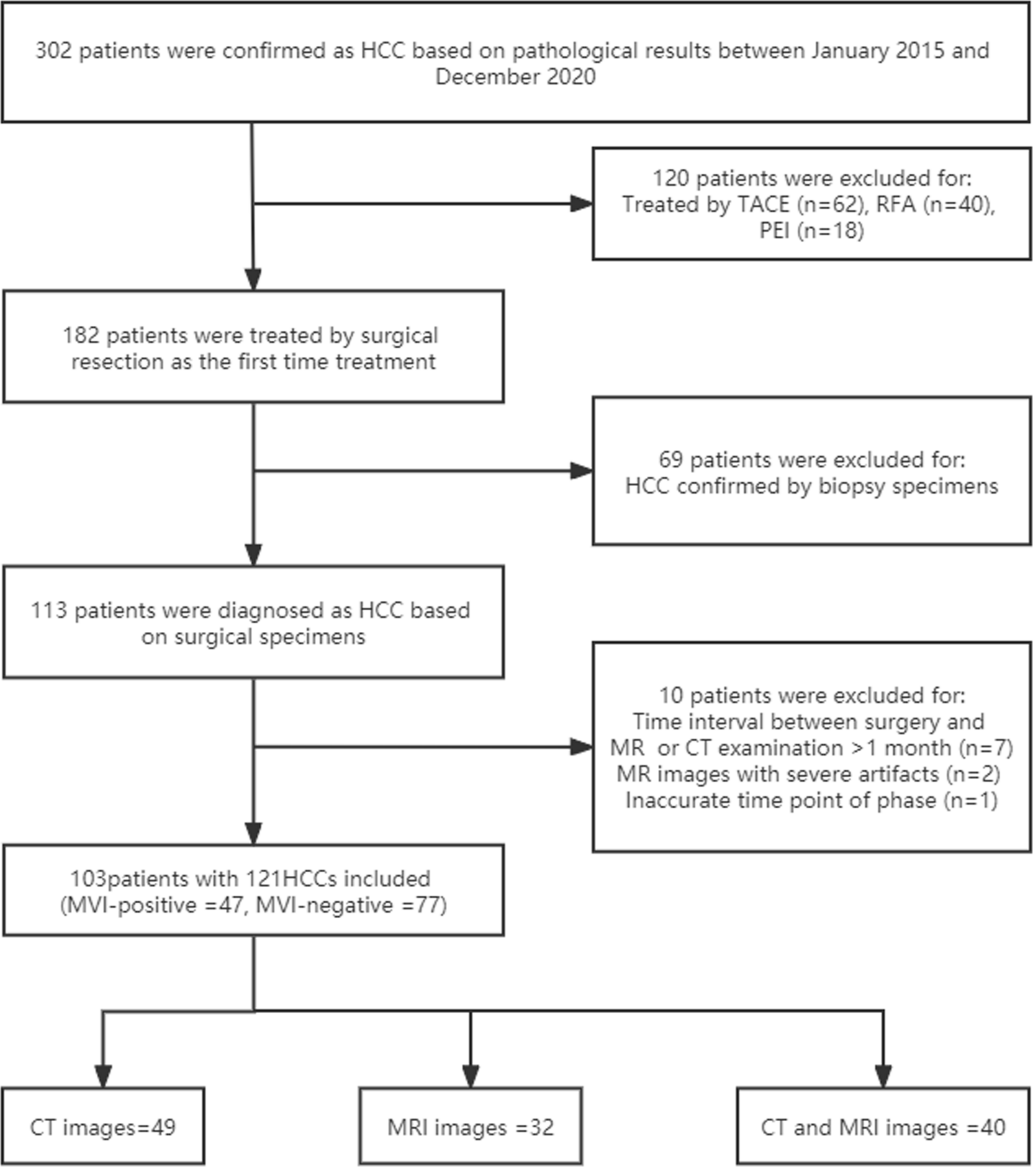
Flow chart for details. HCC, hepatocellular carcinoma; MVI, microvascular invasion; TACE, transcatheter arterial chemoembolization; RFA, radiofrequency ablation; PEI, percutaneous ethanol injection.

This retrospective study was approved by the institutional Human Ethics Committee after the written informed agreement was waived. From January 2015 to December 2020, 302 consecutive patients underwent dynamic contrast-enhanced CT (CE-CT) or/and dynamic contrast-enhanced MRI (DCE-MRI) with other conventional MRI sequences to evaluate HCC in the Department of Radiology, Beijing Friendship Hospital. The inclusion criteria were as follows: (1) four-phasic liver DCE-MRI images were available, including precontrast images and those of the arterial, portal venous, and equilibrium phase, or three-phasic CE-CT images were available, including precontrast images and those of the arterial and portal venous phase (PVP); (2) the pathologic MVI of HCC was obtained by surgical resection; (2) no previous treatment, such as percutaneous ethanol injection, radiofrequency ablation, or transcatheter arterial chemoembolization had occurred. The exclusion criteria were as follows: (1) inaccurate time point of phase; (2) hepatobiliary contrast agent for MRI; (3) an interval between CT or MRI imaging examinations and resection longer than 4 weeks; and (4) prominent artifacts that affected the observation of HCCs. See Flow Chart **Figure 1** for details.

### Image acquisition

Liver CE-CT scans were acquired by various multidetector CT scanners. CT images were obtained before and after administration of contrast agent during the arterial phase (AP), portal venous phase (PVP) and/or equilibrium phase. Among them, plain scan, AP and PVP images were used for analyses. All CE-MRI examinations were performed in 3.0 T scanners. Three MRI sequences were used for analyses: T2-weighted imaging (T2WI), diffusion-weighted imaging (DWI) and T1-weighted imaging (T1WI), including imaging before and after intravenous injection of diethylenetriaminepentaacetic acid (DTPA) at the AP, PVP, and equilibrium phase (EP). Details of imaging acquisition protocols are shown in **Supplementary File 1**.

### Histopathologic MVI diagnosis of HCC

Histopathologic examination for surgical specimens was performed at each site by two experienced pathologists who were unaware of the patient’s radiologic examination results and clinical history. The MVI of HCC was defined as the presence of tumour thrombus in small peritumor vessels (portal vein, hepatic vein or large capsular vesselslined with surrounding liver tissue) only detected under the microscopy. Any differences were resolved by consensus. Details of the histopathologic diagnosis of MVI are provided in **Supplementary File 2**.

### Double-branch multimodality-contribution-aware TripNet based on small samples

**Figure 2:**
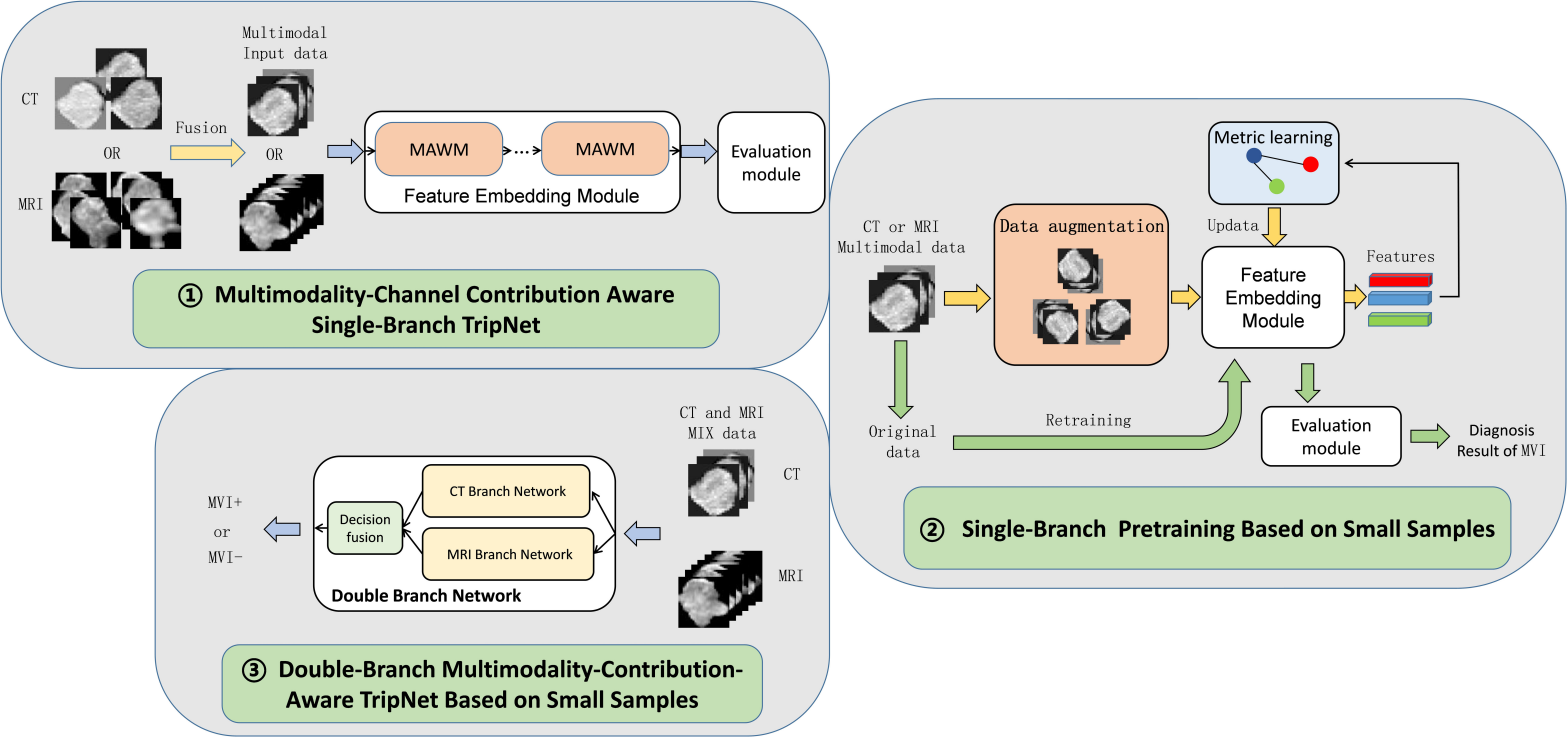
Flow chart of CT&MRI double-branch multimodality-contribution-aware TripNet. MAWM, multimodal channel adaptive weighted modules. The whole model consists of three steps. The first step is to establish Multimodality-Channel Contribution Aware Single-Branch TripNet. The highlight of this step is to integrate the prior knowledge of radiologists by setting the multimodal channel adaptive weighted modules (MAWM). Considering the importance of different modal in clinical diagnosis, the network can adaptively increase important modal features, and the attention to the features of the unimportant modal channels can be reduced. In the second step, based on the first step, a single-branch two-step training strategy is added for small sample problems, including data enhancement and metric learning, and then the CT branch network and MRI branch network are obtained. In the third part, the CT Branch and MRI Branch network are fused and updated with CT and MRI Mixed data, and double-branch Multimodality- contribution-aware TripNet is finally obtained.

Due to the particularity of the data composition with only CT form, only MRI form, and CT&MRI mixed form, the double-branch multimodality-contribution-aware TripNet based on small sample is adopted, and the 2D slice with the largest lesion area in each modality of CT images and MRI images was used for ROI (region of interest) extraction and greyscale normalization. The segmentation boundaries were drawn with ITK-SNAP software (https://www.radiantviewer.com) slice by slice for each volume along the visible borders of the lesion. In order to facilitate understanding, the whole process was divided into three parts. The first part is the establishment of multimodality-channel contribution aware single-branch TripNet, which consists feature embedding module and evaluation module, using pure CT image data and pure MR data respectively. In the feature embedding module, multimodal channel adaptive weighted modules (MAWM) are added to consider the final classification weight of the features in different modal channel dimension that is similar to the prior knowledge of radiologists to consider the importance of different modal in clinical diagnosis. For example, in clinical work, radiologists believe that arterial phase images in CT are more important in the diagnosis of MVI in hepatocellular carcinoma, so we give more weight to arterial phase channels in MAWM. In the second part, single-branch pretraining based on small samples is added for each of the multimodality-channel contribution aware single-branch TripNet to form single branch network including CT branch network and MRI branch network. It consists of two stages, namely, the feature embedding pretraining and the fine-tuning stage of model. The data augmentation and metric learning (22, 23) are added in the feature embedding pretraining to solve small samples problem. In the third part, CT branch network and MRI branch network are weighted and fused according to a certain proportion, and the parameters of the two branches are further updated by mixed CT and MRI data, and finally the double-branch multimodality-contribution-aware TripNet based on small sample is obtained. See **Figure 2** and detailed mechanism and formula in **Supplementary File 3**.

### Statistical analysis

#### Comparison method

To evaluate the effectiveness of the proposed method in the diagnosis and evaluation of hepatocellular carcinoma (HCC) MVI, three groups of comparative tests were conducted including comparison with other deep neural networks, comparison with end-to-end training model and Comparison between double branch network and single branch networks. The whole three comparisons used the same data and same conditions from this study. (1) Comparison with other deep neural networks; we compare the MCAT with other deep neural network (24–26) including two-dimensional convolutional neural networks (2DCNN), residual neural network (ResNet 18), squeeze-and-excitation networks (SENet 18), efficient channel attention network (ECANet 18). Channel attention is introduced from SENet firstly. ECANet and MCAT are optimized on the basis of SENet. ECANet is efficient channel attention and MCAT is Mutli-modals (sequences) contribution aware mechanism. (2) Comparison with end-to-end training model; For the small sample problem, single branch pretraining were added in the model and compare it with the conventional end-to-end training in the same experiment setting. (3) Comparison between double branch network and single branch networks. The double branch network was compared with CT branch network and MRI branch network respectively.

#### Evaluation indicators

The evaluation indexes adopted in this paper are Accuracy, Sensitivity, Precision and F1-Score. The default value 50% was used as the threshold value. The receiver operating characteristic curve (ROC) was drawn for the prediction results of each model, and the area under the curve (AUC) was further used to evaluate the diagnostic quality of MVI. Comparison between AUC values were performed by Z test and P values less than 0.05 were considered statistically significant. Some of the formulas are shown in **Supplementary File 4**.

## Results

A total of 103 patients with 121 pathologically confirmed HCCs were included in this study, including 77 negative for MVI and 44 positive for MVI. The data in this study were composed of 49 CT images (18 positive/31negative), 32 MR images (12 positive/20negative) and 40 mixed images (14 positive/26negative) of HCCs. The demographic and pathological information of the patients is summarized in **Table 1**. Each network training set and validation set were matched in a ratio of 4:1. The data of the training set was expanded by data augmentation in single-branch pretraining based on small samples. The validation set used the original sample data without augmentation. Each network carries out 5 fold cross validation.

**Table 1:** Clinical characteristics of 103 patients and MVI pathological features of HCCs.

	MVI (-)	MVI (+)	*P* value
Number	77	44	
Age(mean,year)	57.23 ± 10.57	57.14 ± 12.82	0.924
Sex			0.583
male	53	28	
female	13	9	
Tumor Diameter(mean,cm)	3.31 ± 2.19	4.31 ± 2.46	0.028
AFP(mean,ng/ml)	362.90 ± 1510.86	2839.88 ± 14174.01	0.540
Cirrhosis			0.072
NO	15	3	
YES	51	34	
Child-Pugh stage			0.586
A	49	31	
B	12	3	
C	5	3	
Location			0.028
Left Lobe	14	18	
Right lobe	63	26	
Pathology classification			0.010
Poorly differentiated	8	10	
Moderately differentiated	66	33	
Well differentiation	5	1	

MVI, microvascular invasion; HCC, hepatocellular carcinoma; AFP, alpha-fetoprotein.

### Comparison with other deep neural networks

**Figure 3:**
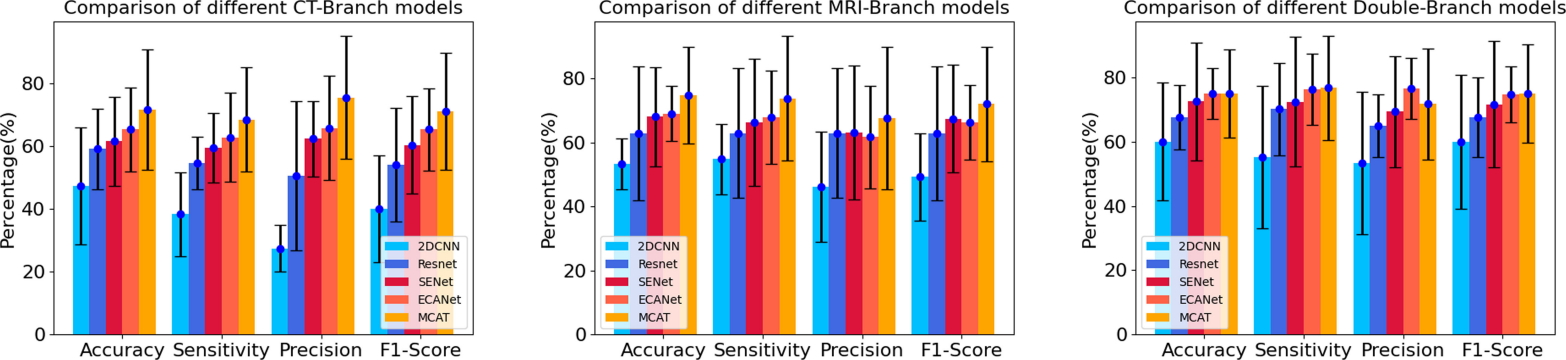
Histograms of accuracy, sensitivity, precision and F1 score of different networks in CT branch, MRI branch and double branch models. The heights of the blue, dark blue, red, orange and yellow histograms represent the average values of accuracy, sensitivity, precision and F1 score in the CT branch, MRI branch and double branch, respectively, using different deep neural networks. The short black line at the top of the histograms represents the standard deviation of of the corresponding mean.

The results are shown in **Table 2**. In the comparison of different deep learning models, no matter in CT branch, MRI branch or double branch, compared with 2DCNN, ResNet, SENet, ECANet, the performance and stability of MCAT are the best. The AUC value of MCAT is the highest in different branches compared with other deep learning models. Especially in the double branch, the AUC values of MCAT compared with 2DCNN, ResNet, SENet and ECANet were 0.83 VS 0.62, 0.67, 0.70 and 0.70, respectively, showing significant differences (p<0.05). In order to display this result more intuitively, we made histogram to show the accuracy, sensitivity, precision and F1-score of different networks of CT branch, MRI branch and double-branch model. See **Figure 3**.

**Table 2:** Diagnostic evaluation performance of different neural networks for HCC MVI (validation data).

	Lesions	MVI (+)	MVI (-)	Neural networks	Accuracy (%)	Sensitivity (%)	Precision (%)	F1 score (%)	AUC	*Z*	*p*
CT branch	49	18	31	2DCNN	47.33 ± 18.66	38.27 ± 13.45	27.44 ± 7.52	39.94 ± 17.04	0.45 ± 0.20	6.242^a^	<0.001^a^
				ResNet	59.11 ± 12.77	54.58 ± 8.37	50.53 ± 23.87	54.11 ± 18.10	0.62 ± 0.12	2.268^a^	0.023^a^
				SENet	61.56 ± 14.17	59.46 ± 11.14	62.36 ± 12.03	60.34 ± 15.54	0.65 ± 0.14	1.228^a^	0.220^a^
				ECANet	65.33 ± 13.43	62.80 ± 14.12	65.71 ± 16.59	65.28 ± 13.10	0.66 ± 0.13	0.946^a^	0.344^a^
				MCAT	71.55 ± 19.22	68.45 ± 16.57	75.46 ± 19.60	71.05 ± 18.56	0.69 ± 0.18	3.395^d^	<0.001^d^
MRI branch	32	12	20	2DCNN	53.33 ± 8.05	54.83 ± 11.07	46.28 ± 17.23	49.27 ± 13.72	0.47 ± 0.23	4.926^b^	<0.001^b^
				ResNet	62.86 ± 20.90	63.00 ± 20.20	63.00 ± 20.20	62.86 ± 20.93	0.63 ± 0.26	1.755^b^	0.079^b^
				SENet	68.10 ± 15.55	66.33 ± 19.84	63.17 ± 20.94	67.51 ± 16.89	0.68 ± 0.26	0.878^b^	0.380^b^
				ECANet	69.05 ± 8.52	68.00 ± 14.54	61.83 ± 16.04	66.37 ± 11.61	0.69 ± 0.19	0.842^b^	0.400^b^
				MCAT	74.76 ± 15.17	73.83 ± 19.50	67.66 ± 22.33	72.08 ± 17.97	0.73 ± 0.19	2.175^d^	0.030^d^
Double branch	40	14	26	2DCNN	60.00 ± 18.37	55.14 ± 22.26	53.33 ± 22.11	59.89 ± 20.77	0.62 ± 0.27	3.953^c^	<0.001^c^
				ResNet	67.50 ± 10.00	70.14 ± 14.33	65.00 ± 9.72	67.59 ± 12.33	0.67 ± 0.10	4.529^c^	<0.001^c^
				SENet	72.50 ± 18.37	72.40 ± 20.14	69.33 ± 17.34	71.63 ± 19.76	0.70 ± 0.21	2.836^c^	0.005^c^
				ECANet	75.00 ± 7.91	76.31 ± 11.04	76.48 ± 9.47	74.68 ± 8.74	0.70 ± 0.19	2.984^c^	0.003^c^
				MCAT	75.00 ± 13.69	76.66 ± 16.16	71.66 ± 17.34	74.93 ± 15.19	0.83 ± 0.20		

MVI, microvascular invasion; HCC, hepatocellular carcinoma; 2DCNN, two-dimensional convolutional neural networks; ResNet, residual neural network; SENet, squeeze-and-excitation Networks; ECANet, efficient channel attention network; MCAT, multimodality-contribution-aware TripNet; AUC, the areas under the receiver operating characteristic curves; a, comparison AUC value of CT branch between 2DCNN, ResNet, SENet, ECANet and MCAT respectively; b, comparison AUC value of MRI branch between 2DCNN, ResNet, SENet, ECANet and MCAT respectively; c, comparison AUC value of double branch between 2DCNN, ResNet, SENet, ECANet and MCAT respectively; d, comparison AUC value of MCAT model between CT branch, MRI branch and double branch respectively.

### Comparison with end-to-end training model

**Figure 4:**
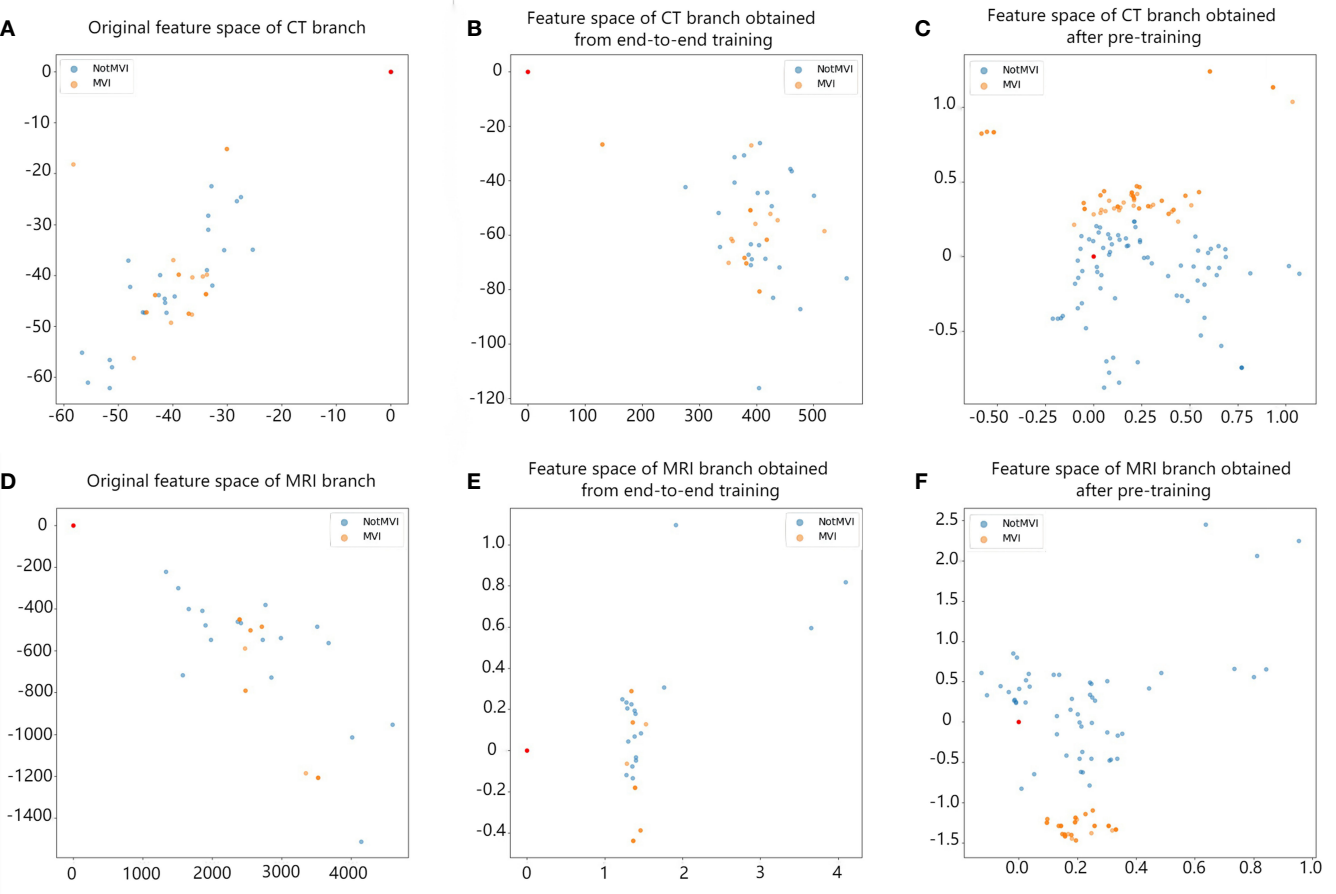
The comparison between the feature space obtained by single-branch pretraining based on small sample and the feature space obtained by end-to-end training. Each point in the figure represents a lesion. Blue represents MVI-negative lesions, and orange represents MVI-positive lesions. Panels **(A, D)** represent the original feature space of the CT branch and MRI branch. Blue and orange points are irregularly mixed together. Panels **(B, E)** show the feature space of the CT branch and MRI branch after end-to-end training. Blue and orange points start to gather, but they are still mixed together. Panels **(C, F)** show the feature space of the CT branch and MRI branch after single-branch pretraining based on small samples. Points of the same colour begin to gather, and blue points and orange points are basically separated, which improves the ability to distinguish between negative and positive MVI.

In the same experiment setting, comparison between end-to-end training and single branch pretraining based on small samples in different branches have been carried out. The results are shown in **Table 3**. The AUC value of model with single branch pretraining based on small samples is higher than model with end-to-end training in CT branch, MRI branch or double branch (0.62 vs 0.69, 0.68 vs 0.73, 0.65 vs 0.83, respectively). And among them the AUC values in the CT branch and double branch were statistically different (Z=2.41, p=0.016 and Z=2.54, p=0.010, respectively). In order to intuitively demonstrate the effectiveness of the single branch pretraining based on small samples, we also visualized the feature embedding space of the CT branch and MRI branch obtained by the two training methods, as shown in **Figure 4**.

**Table 3:** Comparison between end-to-end training and single-branch pretraining on the performance of the final model.

	Training methods	Accuracy (%)	Sensitivity (%)	Precision (%)	F1 score (%)	AUC	*Z*	*p*
CT branch	end-to-end training	63.33 ± 16.05	62.61 ± 10.31	66.56 ± 16.57	61.64 ± 17.04	0.62 ± 0.09	2.408^a^	0.016^a^
	single-branch pretraining	71.55 ± 19.22	68.45 ± 16.57	75.46 ± 19.60	71.05 ± 18.56	0.69 ± 0.18		
MRI branch	end-to-end training	68.09 ± 15.55	66.33 ± 19.84	63.17 ± 20.94	67.51 ± 16.89	0.68 ± 0.26	0.874^b^	0.382^b^
	single-branch pretraining	74.76 ± 15.17	73.83 ± 19.50	67.66 ± 22.33	72.08 ± 17.97	0.73 ± 0.19		
Double branch	end-to-end training	65.50 ± 16.96	70.14 ± 19.82	62.29 ± 22.95	66.17 ± 21.31	0.65 ± 0.4	2.547^c^	0.011^c^
	single-branch pretraining	75.00 ± 13.69	76.66 ± 16.16	71.66 ± 17.34	74.93 ± 15.19	0.83 ± 0.20		

AUC, the areas under the receiver operating characteristic curves; ^a^, Comparison AUC value of CT branch between using end-to-end training and using single-branch pretraining; ^b^, Comparison AUC value of MRI branch between using end-to-end training and using single-branch pretraining; ^c^, Comparison AUC value of double branch between using end-to-end training and using single-branch pretraining; end-to-end training, without single-branch pretraining; single-branch pretraining= for solving small sample problem to create including feature embedding pretraining (data augmentation and metric learning) and the fine-tuning stage of model.

### Comparison between double branch network and single branch networks

**Figure 5:**
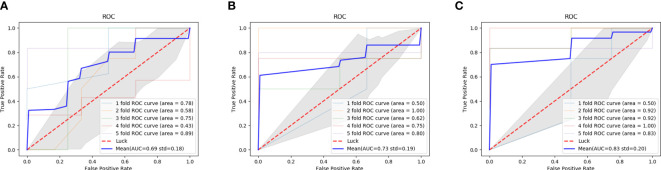
ROC curves of the performance of different diagnostic branch models in the 5-fold cross test. **(A)** the AUC of the CT branch; **(B)** the AUC of the MRI branch; **(C)** the AUC of the MIX branch; ROC, receiver operating characteristic curve; AUC, the area under the receiver operating characteristic curve.

The comparison results between double branch network and single branch networks are shown in the **Table 2**. ROC curves and AUC values of CT branch network, MRI branch network and double branch network in the 5-fold crossover experiment were plotted and calculated, and the experimental results are shown in **Figure 5**. By comparing the average AUC value of different branch network, the average AUC value of the double branch network was significantly higher than that of the CT branch network or MRI branch, network with Z=3.39, p<0.001 and Z=2.18, p=0.029, respectively. But there is no significant difference in the average AUC value between CT branch network and MRI branch network (Z=0.934, p=0.350).

**Figure 6:**
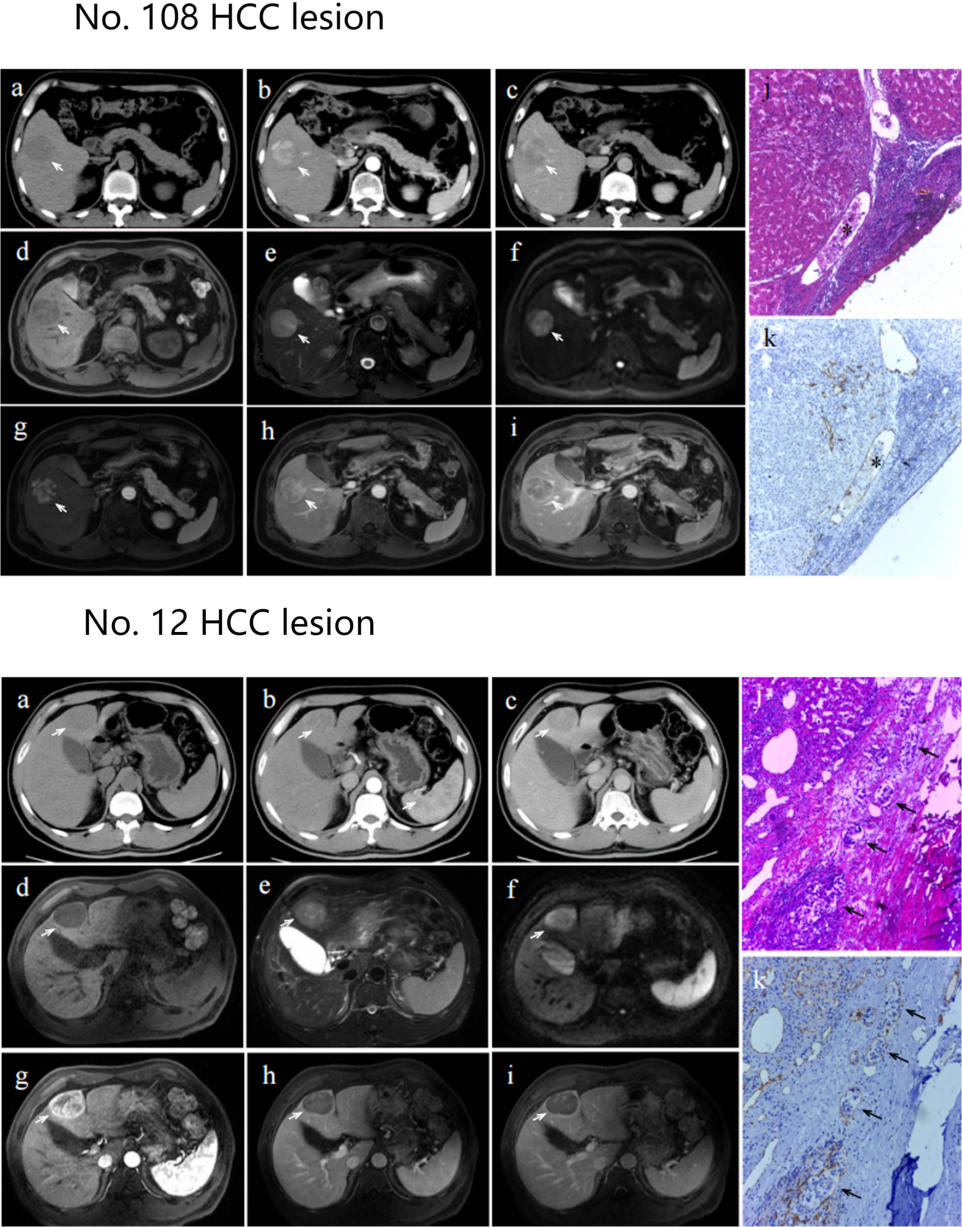
Axial CT images, axial MRI images and pathological section maps of No. 108 and No. 12 HCC lesion Axial CT images **(A)**: plain scan; **(B)**: arterial phase; **(C)**: portal venous phase) and axial MRI images **(D)**: T1-weighted imaging; **(E)**: T2-weighted imaging; **(F)**: diffusion weighted imaging; g: arterial phase; **(H)**: portal venous phase; **(I)**: venous phase. The lesion is indicated by a white arrow. Pathological section maps with HE staining **(J)** and CD31 staining **(K)** showed positive microvascular invasion, as indicated by black asterisks or black arrows.

The diagnostic results of two cases with MVI positive lesions (No.108 and No. 12) in different branch networks (CT branch network, MRI branch network and double branch network) were presented. The double branch network and MRI branch network of lesion No. 108 were correctly diagnosed, while the CT branch network was incorrectly diagnosed (the probability of predicting positive MVI was 0.7, 0.7 and 0.1, respectively). The double branch network and CT branch network of lesion No. 12 were correctly judged, while the MRI branch network was incorrectly judged (the probability of predicting positive MVI was 0.6, 0.8 and 0.4, respectively). When the probability of predicting positive MVI was greater than 0.5, the network classified it as positive. CT and MRI images and pathological results of these two lesions are shown in **Figure 6**.

## Discussion

In comparison to other deep neural networks, our study shows that the proposed double-branch MCAT model could significantly improves the prediction accuracy of MVI in HCC by the use of the modal (sequences) attention schemes and fusion of CT and MRI images based on small samples.

SeNet (25) is proposed as a representative channel attention mechanism based on ResNet and CNNs in 2017. It proposes a novel architectural unit, which is termed the “Squeeze-and-Excitation” (SE) block, calculates the weighted features by focusing on the importance of different channels of feature vectors. Considering that different channel-wise features have various contributions to the different clinical issues such as diagnosis of HCC or evaluation of MVI in HCC, some studies have attempted to apply SE block in the field of medical image analysis. In Chen et al. (27) study, a SE block was introduced into traditional CNN which achieved a good performance improvement in classification of benign and malignant pulmonary nodules with a max AUC value of 0.930. In Jia et al. (28) study to the diagnosis of HCC pathologic grades, a SE block was combined with residual calculation Block to calculate the contribution degree of multiple MRI sequences such as T2WI and T1WI with a purpose to inhibit the influence of non-important MRI sequences while paying attention to important MRI sequence. However, although several studies (19–21) were focused on the prediction of MVI in HCC by using traditional CNN or ResNet, few study has applied SE block to improve the prediction accuracy. ECANet (26) and MCAT are optimized on the basis of SENet. ECANet is efficient channel attention and MCAT is Mutli-modals (sequences) contribution aware mechanism based on modal (sequences) attention. ECANet is currently mainly used in computer vision for image classification and segmentation, such as electrocardiogram classification (29) and image reconstruction (30). In our study, the double-branch MCAT consists of MAWM (multimodal channel adaptive weighted modules), an improved version of SE blocks, as the main network for the first time to predict MVI of HCC, was better performance than other neural networks in internal verification. Comparing to similar studies Zhang Y et al. (20) using 3D CNN and Liu, S et al. (21) using ResNet 18, the performances were also significantly improved with AUC value of 0.83 vs. 0.72 and 0.75.

As popular approaches to solve the small-sample size problems, both data augmentation and metric learning were also used in the proposed MCAT model to improve the prediction accuracy (28, 31). Theoretically, data augmentation can enrich the sample diversity and solve the problem of small samples at the data level. while the measurement of metric embedding loss can make the obtained feature space more discriminable and solve the problem of small samples at the feature level (22, 23). In our study, we used both data augmentation and metric learning to solve small sample problems with remarkable results. Jingwei et al. (19) included a sample size of 750 cases in their study, in which the deep learning model ResNet18 without data augmentation and metric learning. The AUC value of CE-CT and EOB-MRI were 0.734 and 0.802, respectively. The total number of samples in our study was 121, and the data volume of the double branch was only 40 cases, with an AUC value of 0.83, which is higher than the previous results of a large sample size. This indicates that data augmentation and metric learning could be a solution to the problem of small sample size without affecting the results. Moreover, our study shows that using single branch pretraining can intuitively improve the diagnostic evaluation performance of the model compared with end-to-end training, effectively reduce the standard deviation of each evaluation index, and make the model performance obtained by training more excellent and stable. In computing, the power of 10 is an order of magnitude, and 100 and 1000 are small sample sizes for a computer. In medical imaging, more than 100 well-annotated images are difficult to obtain. Therefore, the application of data augmentation and metric learning can greatly solve the problem of small sample sizes in the medical field.

Additionally, in our study, the double branch of CT and MRI images was more efficient than the single branch of CT or MRI images alone, with AUCs of 0.83, 0.69 and 0.73, respectively. A possible explanation is that CT and MRI are different imaging techniques and have different advantages in evaluating MVI. Jingwei Wei et al. (19) established ResNet18 models based on CE-CT and EOB-MRI images and concluded that the model based on EOB-MRI had a better effect than the model based on CE-CT with AUC values of 0.802 and 0.734, respectively. In addition, Hu et al. showed that CT is superior to MRI in evaluating tumour margins (13). CT and MRI have their own imaging characteristics. In contrast, CT has higher spatial resolution, while MRI has higher tissue resolution. Therefore, CT and MRI images may also provide texture features in different directions for deep learning models, and the simultaneous uptake of these features can improve diagnostic efficiency. Moreover, our model can accept different imaging images to predict MVI of HCC, such as CT, MRI, or both. This may have broader clinical applicability. For various reasons, we cannot guarantee that patients will be able to choose imaging modalities as we require.

Our study also has some limitations. First, the number of HCCs in the present study is limited, which may influence the generalization of the deep learning model. However, we adopted data augmentation and metric learning to solve this problem. Second, MVI grade was not considered in the MVI-positive group. In addition, other contrast agents or Gd-EOB-DPTA-enhanced MRI images in the hepatobiliary phase were not compared or evaluated in this study, and we will continue to study them in the future.

In conclusion, our study indicates that double branch MCAT based on small samples can improve the effectively compared with other deep neural networks, providing a solution for scenarios such as small-sample deep learning and fusion of different imaging technologies.

## Data availability statement

The original contributions presented in the study are included in the article/**Supplementary Material**. Further inquiries can be directed to the corresponding authors.

## Author contributions

Study concept and design: DY, ZY. Acquisition of data: YD, XJ. Analysis and interpretation of data: DY, ZY,YD, XJ. Drafting of the manuscript: DY, YD. Critical revision of the manuscript for important intellectual content: GY, JH, HX, AR, ZW. Administrative, technical, or material support, study supervision: ZW. All authors have made a significant contribution to this study and have approved the final manuscript. All authors contributed to the article and approved the submitted version.

## Funding

This work is supported by the National Natural Science Foundation of China (No. 82071876, 61871276), Beijing Municipal Administration of Hospitals’ Youth Programme (No. QML20200108), Heilongjiang Provincial Natural Science Foundation of China (No. QC2018107).

## Acknowledgments

The authors would like to express our enormous appreciation and gratitude to all participants.

## Conflict of interest

The authors declare that the research was conducted in the absence of any commercial or financial relationships that could be construed as a potential conflict of interest.

## Publisher’s note

All claims expressed in this article are solely those of the authors and do not necessarily represent those of their affiliated organizations, or those of the publisher, the editors and the reviewers. Any product that may be evaluated in this article, or claim that may be made by its manufacturer, is not guaranteed or endorsed by the publisher.
