# Erratum: Can a proposed double branch multimodality–contribution–aware TripNet improve the prediction performance of the microvascular invasion of hepatocellular carcinoma based on small samples?

**DOI:** 10.3389/fonc.2022.1092191

**Published:** 2022-12-01

**Authors:** Frontiers Production Office

**Affiliations:** Frontiers Media SA, Lausanne, Switzerland

**Keywords:** hepatocellular carcinoma, CT, deep learning, MRI, microvascular invasion

Due to a production error, the figures included in the article were not listed in the correct order. Figure 3 was included as Figure 4, Figure 4 was included as Figure 3, Figure 5 was included as Figure 6, and Figure 6 was included as Figure 5.

The publisher apologizes for this mistake. The original version of this article has been updated.

