# Supplementary material for: Can a proposed double branch multimodality-contribution-aware TripNet improve the prediction performance of the microvascular invasion of hepatocellular carcinoma based on small samples?
Source: Front Oncol. 2022 Oct 24;12:1035775. doi: 10.3389/fonc.2022.1035775 (PMC9640917; doi:10.3389/fonc.2022.1035775)
Supplement: Supplementary file 1 [file DataSheet_1.docx]

Supplementary Files

# Supplementary Data

**File 1:** **Detailed descriptions of imaging acquisition protocols**

***(1).*** ***Magnetic******resonance*** ***imaging******(MRI)*** ***protocols***

Gadolinium-DTPA (Gd-DTPA) enhanced MRI imaging was performed using various 3.0T MRI scanners with16-channel phased-array body coil. The routine MRI sequences included: i) spin-echo T2-weighted imaging; ii) diffusion-weighted sequence (*b* values: 0 s/mm^2^ and800 s/mm^2^), and iii) a fat-suppressed three-dimensional (3D) gradient-echo T1 weighted sequence before and after intravenous injection of Gd-DTPA at the arterial phase ([AP] bolus triggering, 30s), portal venous phase ([PVP] 60-70s), equilibrium phase (3 min). A standard dose (0.1 mmol/kg) of Gd-DTPA (Magnevist, Bayer-Schering Pharma, Berlin, Germany) was injected at a rate of 2.0 ml/s, with an immediately followed 20 ml saline ﬂush through an antecubital venous catheter with a dual power injector. Detailed imaging parameters of Gd-DTPA MRI are shown in Table 1.

***(2).*** ***Computed******tomography*** ***(CT)******protocols***

Multiphasic contrast-enhanced CT (CE-CT) was performed using various multi-detector CT scanners including Philips ICT, GE Revolution CT and GE Discovery CT750HD. All patients received a nonionic contrast medium (Iopamidol, 370 mg of iodine per milliliter [Iopamidol 370; Bracco Imaging, Milan, Italy]) at a dose of 1.4 mL (518 mg of iodine) per kilogram of body weight at a rate of 3.0-4.0 ml/s. Prewarmed contrast material was administered intravenously at a rate of 2-2.5 ml/s through an antecubital vein. After acquisition of an anteroposterior digital scout radiograph, patients were scanned craniocaudally from the dome of the liver to the iliac crest before and after intravenous contrast medium administration. Images were obtained during the AP (bolus tracking, 15-20 s after trigger thresholds ranging between 170-182 Hounsfield units [HU] were reached in the supra-coeliac abdominal aorta), PVP (70-80s after the administration of contrast media), and equilibrium phase (180s after the administration of contrast media). Detailed imaging parameters of multiphasic CE-CT are shown in Table 2.

**File 2. Details of histopathologic diagnosis for microvascular invasion (MVI)**

MVI was defined as the presence of tumor thrombi within small peritumoral vessels (branches of portal vein, hepatic vein, or a large capsular vessel of the surrounding hepatic tissue lined by endothelium) detected only on micros- copy [1; 2]. To ensure adequate and reliable detection of MVI, a 7-site sampling procedure was performed at each center. All the HCC specimens were cut apart along the maximal tumor section and were then sliced into serial 1-cm thick sections parallel to the maximal tumor section, with the total number of sections determined by the tumor size. Afterwards, one piece of tissue was each sampled at the transition area between tumor and surrounding liver tissues with a ratio of 1:1 at 12, 3, 6 and 9 o’clock from the less bleeding and necrotic sections. One piece was sampled from the tumor area free from bleeding and necrosis, and one piece each was sampled respectively from proximal (≤1.0 cm to the tumor) and distant liver parenchyma (> 1 cm away from the tumor). Thus, at least 7 tissue blocks were sampled. These tissue blocks were first sampled crossing the maximal tumor section. If the number of tissue block acquired from this section could not reach more than 7, the remaining tissue blocks were sampled from sections elsewhere of the specimens. Two experienced pathologists independently reviewed all specimen slices for each tissue block to determine the presence of MVI at each site. If MVI was detected in any one of all tissue clocks, MVI was reported as positive for this patient.

**File 3.** **Details of** **Double-Branch Multimodality-Contribution-Aware TripNet Based on Small Samples**

After data preprocessing, the whole model is divided to three steps. The first step is to establish multimodality-channel contribution aware single-branch TripNet using pure CT image data and pure MRI data respectively. In the second step, based on the first step, single-branch pretraining is added for small sample problems to obtain CT branch network and MRI branch network. In the third part, CT branch network and MRI branch network are fused and updated with CT and MRI mixed data, and double-branch multimodality- contribution-aware TripNet based on small samples is finally obtained, namely double branch network. Details are shown as follow.

***3.1 Data Preprocessing***

Tumour segmentation was manually and independently performed by two radiologists (with at least 3 years of HCC diagnosis experience) for the phases of the CT and MRI data, and reviewed by another radiologist with 10 years of HCC diagnosis experience. The segmentation boundaries were drawn with ITK-SNAP software (https://www.radiantviewer.com) slice by slice for each volume along the visible borders of the lesion. The 2D slice with the largest lesion area in each modality of CT images and MRI images was used for ROI (region of interest) extraction and greyscale normalization. The median lesion size of 64 was selected according to the size of the extracted HCC lesion area, and then the ROI was resized to 64*64. The ROI data of 2D lesions in each modality of CT and MRI were spliced along the channel dimension to obtain the multimodal fusion data of CT and MRI. The multimodal fusion data were used to train the CT branch and MRI branch. The input data sizes of these two branches were 64*64*3 and 64*64*6, respectively.

***3.2 Multimodality-Channel Contribution Aware Single-Branch TripNet***

The overall structure of multimodality-channel contribution aware single-branch TripNet consists of feature embedding module and evaluation module, as shown in Fig.1. The feature embedding module calculates the multimodal weighted fusion features by assigning different weights to the image information of different modal. The evaluation module is responsible for MVI positive/negative diagnostic evaluation based on the multimodal weighted fusion features output by the feature embedding module.

1. ***Feature Embedding Module***

Feature embedding module consists of a 3 x 3 convolution and three multimodal feature extraction layers, each modal feature extraction layer contains three multimodal channel adaptive weighted modules (MAWM). Each MAWM by considering the final classification weight of features in different modal-channel dimension, making the network adaptive learning important modal features, reducing attention to the features of unimportant modal is consistent with the prior knowledge of radiologists to consider the importance of different modes in clinical diagnosis.

Each MAWM squeezes the input feature vectors $F\in R^{H\times W\times N*C}$ along the channel dimension. The squeeze method adopts the global average pooling (GAP) operation to obtain the squeezed fusion features $F_{1}\in R^{1\times1\times C}$, where $H$ and $W$ respectively represent the height and width of features, $N$ represent the number of modals contained in the multimodal feature $F$ and $C$ represent the number of channels of each modal after feature extraction of multimodal fusion data. The calculation formula of the squeeze operation is as follows:

|  | $F_{1}=GAP(F)=\frac{1}{H\times W}\sum_{i=1}^{H} \sum_{j=1}^{W} F_{C}(i,j)$ | (1) |
| --- | --- | --- |

Second, in the excitation operation, this paper adopts one learnable parameters $W_{1}\in R^{N*C\times N}$,bias term $b$ and the ReLU activation function to learn the correlation between the contribution of each modal to the classification task, as shown in Formula (2).

|  | $F_{2}=\delta(W_{1}F_{1}+b)$ | (2) |
| --- | --- | --- |

$F_{2}\in R^{1\times1\times C}$ represents the learned contribution vector, where $\delta(\cdot)$represents the ReLU activation function. Finally, the final multimodal weighted fusion vector $F_{3}\in R^{H\times W\times C}$is obtained by reweighting the normalized contribution vector $F_{2}$ with the original feature map in the modal dimension, and the calculation formula is as follows:

|  | $F_{3}=F\otimes soft\max(F_{2})$ | (3) |
| --- | --- | --- |

$\otimes$represents the dot product in modal dimensions and assigns corresponding weights to each feature channel to pay attention to the features of important modal channels and suppress the feature information of unimportant modal channels. $\mathrm{soft}\max(\cdot)$represents the normalization function such that the sum of the weights of all modals is 1.

Finally, the weighted multimodal fusion vector $F_{3}$, which contains the information of CT or MRI multimodal sequences and is combined with the contribution weight learned by the model. The weighted fusion vector represents the multimodal information after reassigning different attention, which is consistent with the prior knowledge that radiologists consider the importance of different models in clinical diagnosis.

***(2) Evaluation Module***

The evaluation module is composed of a three-layer MLP (input layer-hidden layer-output layer). The multimodal weighted fusion features of anchor samples extracted from the multimodal channel adaptive weighting module are input into the MLP, and the positive/negative classification probability of MVI is output through the full-connection layer and $\mathrm{softmax}$ function. The formula is as follows:

|  | $p_{j}=e^{g(x)_{j}}/\sum_{i=1}^{N} e^{g(x)_{i}}$ | (4) |
| --- | --- | --- |

$p_{j}$ represents the probability that sample $x$ belongs to class $j$, $g(\cdot)$represents the MLP, and $N$represents the dimension of the output vector of the MLP, i.e., the number of categories to be classified, which is set to 2 in this study.

The parameter update of the classification module is based on the multi classification cross-entropy loss constraint, and the formula is as follows:

|  | $l_{cross}=-\sum_{i=1}^{N} y_{i}\log(p_{i})$ | (5) |
| --- | --- | --- |

$y$represents the one-hot label of the sample $x$. N denotes the number of classes to be classified, and $p_{i}$ represents the probability that the sample belongs to the $i$th class.

***3.3 Single-Branch Pretraining Based on Small Samples***

In order to solve the problem that it is difficult to conduct end-to-end training of deep neural network with a small amount of data, this paper adds a single-branch pretraining based on small samples for the multimodality-channel contribution aware single-branch TripNet to form CT branch network and MRI branch network. The pretraining process consists of two stages, namely, feature embedding pretraining and the fine-tuning stage of model.

***(1）Feature Embedding Pretraining Stage***

In the feature embedding pretraining stage, this paper adopts the pretraining method based on small sample learning to preliminatively learn the parameters of feature embedding module, so as to obtain a highly discriminable feature embedding space. This pretraining method can alleviate the risk of overfitting at the data layer and feature layer, reduce the contingency of random initialization network to the problem of small samples, and provide a good pretraining parameter for the subsequent learning of evaluation module and the overall fine-tuning of the network.

**Data Augmentation**: At the data layer, to simulate the possible forms of clinical cases when training the feature extraction module and enrich the diversity of samples, 7 geometric data-augmentation operations are used to the training data to expand the amount of data, including clockwise rotation 90°,180°,270°, random up and down flip, random left and right flip, diagonal transpose and subdiagonal transpose.

**Metric Learning**: At the feature layer, in order to obtain a more distinguishable feature space, and solve the problem of extremely similar samples from different classes to distinguish, in this paper, the idea of metric learning optimization feature space is adopted, that is, the training data after data augmentation is sent to the feature embedding network to obtain the embedding features of each sample, and metric-based constraints is used to optimize the parameters of the feature embedding module. Because of involving only single domain single task, this article chooses parameterless triples loss, [3-5] as optimization constraint of feature extraction module. The optimization process of the triplet loss on the feature space is shown in Fig 2. The idea is that by selecting triplet tuples in a data batch, calculate the similarity between samples of the same class and the difference between samples of different classes to learn the discriminant feature space, as shown in formula (6).

|  | $L=\max((d(a,p)-d(a,n)+m\arg in),0)$ | (6) |
| --- | --- | --- |

$a$,$p$,$n$ represent the anchor sample, positive sample and negative sample in the triplet tuple, respectively.$(a,p)$ represents sample pairs from the same class, $(a,n)$ represents sample pairs from different classes. $d(\cdot)$ represents the distance function used to calculate the similarity between two sample feature vectors, and the Euclidean distance is used in this paper. $\mathrm{margin}$ represents a boundary hyperparameter.

***（2）The Fine-tuning Stage of The Model***

In the fine-tuning stage of the model, in order to make the classifier more closer to the real clinical samples, this paper uses the original sample data without augmentation to learn the classifier. Firstly, the pretrained feature embedding module parameters are loaded. After the feature embedding module, a multi-layer perceptron is randomly initialized as a evaluation module. The original sample data and cross entropy loss are used to learn the evaluation module and fine-tune the pretrained feature embedding module, as shown in formula (7), so as to obtain the final pretraining parameters of CT or MRI image branches.

|  | $L=-\sum_{i=1}^{N} y_{i}\log(p_{i})$ | (7) |
| --- | --- | --- |

$L$ Represents the cross entropy loss between the output prediction result of the evaluation module and the real label of the sample, y represents the one-hot label coding of the sample category $x$, $N$ represents the number of classes, and $p_{j}$ represents the probability that the sample belongs to the category $j$.

***3.4 Double-Branch Multimodality-Contribution-Aware TripNet Based on Small Samples***

After completing the above steps, the initial parameters of two single branches are loaded, and the output results of the two single branches are weighted and fused according to a certain proportion. The fusion ratio adopted in this paper is 1:1, that is $\mathrm{Re}sult_{Total}=0.5*\mathrm{Re}sult_{ct}+0.5*\mathrm{Re}sult_{mr}$. The two branch parameters are further updated by CT and MRI mixed data. In the process of parameter updating, the result of fusion decision is constrained by$\mathrm{Re}sult_{Total}$and the cross entropy loss calculated by one-hot encoding of the real label and $\mathrm{Re}sult_{Total}$.

Finally, the double-branch multimodality-contribution-aware tripnet based on small samples was obtained, namely double branch network, which could not only diagnose the MVI degree of hepatocellular carcinoma region in individual CT images or MR images, but also comprehensively diagnose the MVI degree of hepatocellular carcinoma region in two kinds of images, increasing the clinical applicability.

**File 4: Some of the Statistical Analysis formulas as follows.**

|  | $Accuracy=\frac{TP+TN}{TP+TN+FP+FN}$ | (8) |
| --- | --- | --- |
|  | $Sensitivity=\frac{TP}{TP+FN}$ | (9) |
|  | $Precision=\frac{TP}{TP+FP}$ | (10) |
|  | $F_{1}=\frac{2\times P\text{recision}\text{×}Sensitivity}{P\text{recision+}Sensitivity}$ | (11) |

$TP$, $TN$, $FP$, and $FN$ represent the number of true positive samples, true negative samples, false positive samples and false negative samples in the predicted results, respectively. Accuracy reflects the proportion of correctly predicted samples in all samples. Sensitivity reflects the proportion of correctly predicted positive samples in total true positive samples, also known as the true positive rate. Precision reflects the proportion of correctly predicted positive samples in total predicted positive samples, ($1-Precision$) also known as the false positive rate. The F1 score is the result of weighing the comprehensive output of sensitivity and precision, and its value range is [0,1]. The closer the value of the F1 score is to 1, the better the output performance of the model.

**References**

1 Roayaie S, Blume IN, Thung SN et al. A system of classifying microvascular invasion to predict outcome after resection in patients with hepatocellular carcinoma. Gastroenterology, 2009,137:850-855

2 Rodriguez-Peralvarez M, Luong TV, Andreana L, Meyer T, Dhillon AP, Burroughs AK. A systematic review of microvascular invasion in hepatocellular carcinoma: diagnostic and prognostic variability. Annals of surgical oncology, 2013,20:325-339

3 Iguchi T, Shirabe K, Aishima S, et al. New pathologic stratification of microvascular invasion in hepatocellular carcinoma: predicting prognosis after living-donor liver transplantation[J]. Transplantation, 2015, 99(6): 1236-1242.

4 Hermans A, Beyer L, Leibe B. In defense of the triplet loss for person re-identification[J]. arXiv preprint arXiv:1703.07737, 2017.

5 Uzhinskiy A V, Ososkov G A, Goncharov P V, et al. One-shot learning with triplet loss for vegetation classification tasks[J]. Компьютерная оптика, 2021, 45(4): 608-614.

# Supplementary Figures and Tables

## Supplementary Tables

**Table 1 Detailed** **imaging** **parameters** **of Gd-DTPA MRI**

| **Sequence** | **MRI** **unit** | **TR**  **(ms)** | **TE**  **(ms)** | **Flip** **angle** **(°)** | **Matrix** | **FOV**  **(mm^2^）** |
| --- | --- | --- | --- | --- | --- | --- |
| T2-weighted imaging | 3.0T Siemens Prisma  3.0T GE Healthcare GE 750w  3.0 T Philips Ingenia | 2160  6315  4918 | 100  85  106 | 160  150  160 | 320x288  288x244  288x224 | 433x433  360x280  285x380 |
| Diffusion-weighted imaging | 3.0T Siemens Prisma  3.0T GE Healthcare GE 750w  3.0 T Philips Ingenia | 5600  3000  5100 | Minimum  Minimum  55 | 90  90  90 | 100x76  128x128  128x128 | 380x289  360x380  285x380 |
| Dynamic T1-weighted imaging | 3.0T Siemens Prisma  3.0T GE Healthcare GE 750w  3.0 T Philips Ingenia | 3.95  4.1  3.47 | Minimum  Minimum  1.36 | 9  15  10 | 352x256  288x172  320x216 | 400x296  380x300  308x380 |

TR=repetition time; TE=echo time; ST=section thickness; FOV=field of view.

**Table 2 Detailed** **imaging** **parameters** **of** **multiphasic** **CE-CT**

|  | Philips ICT | GE Revolution | GE Discovery CT750HD |
| --- | --- | --- | --- |
| No. of channels | 256 | 256 | 256 |
| Tube voltage (kV) | 120 | 120 | 120 |
| Tube current (mA) | 420 | 450 | 450 |
| Rotation period (s) | 0.5 | 0.5 | 0.6 |
| Helical pitch | 0.991 | 0.992 | 0.984 |
| Acquisition time (s) | 2-6 | 2-6 | 2-6 |
| Section thickness (mm) | 1-5 | 1.25-5 | 1-5 |
| Intersection gap | 0 | 0 | 0 |
| Reconstruction kernel | soft tissue | standard | standard |
| Volumetric CT dose(mGy) | 14.06 | 14.17-17.57 | 17.13 |

## Supplementary Figures

**Fig 1. Multimodality-Channel Contribution Aware Single-Branch TripNet for CT or MRI images**


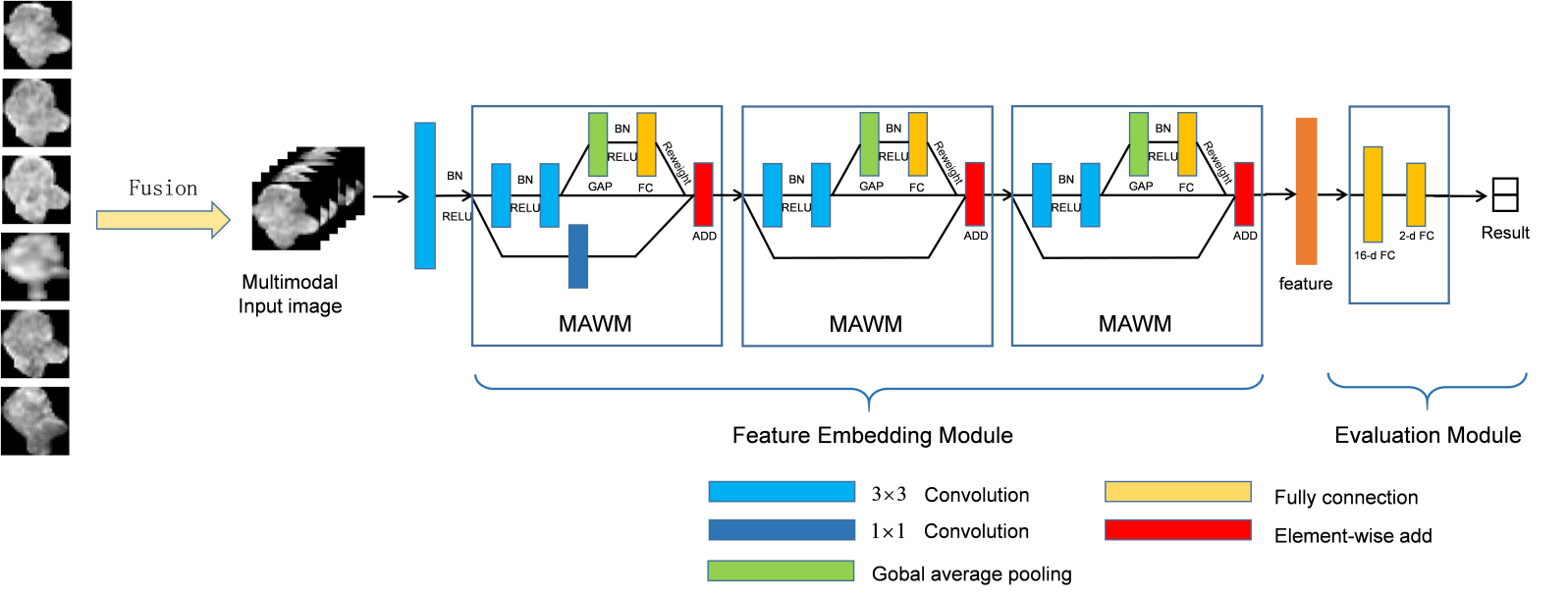


Note. MAWM = multimodal channel adaptive weighted module.

Only one multi-modal feature extraction layer structure is displayed in the feature embedding module, and the remaining two layers have the same structure.

**Fig 2. The optimization process of the triplet loss on the feature space**


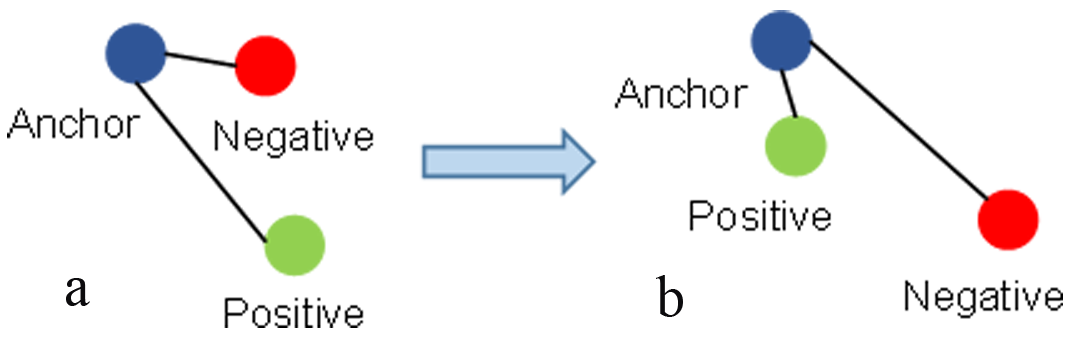


Note. a = original sample triplet characteristics; b = optimized sample triplet characteristics

By comparing the distance between the anchor sample, positive sample and negative sample in the triplet, the distance between the anchor sample and positive sample in the embedding space is as small as possible, and the distance between the anchor sample and negative sample is as large as possible, so as to increase the cohesion between like samples and the separation between different like samples.

**
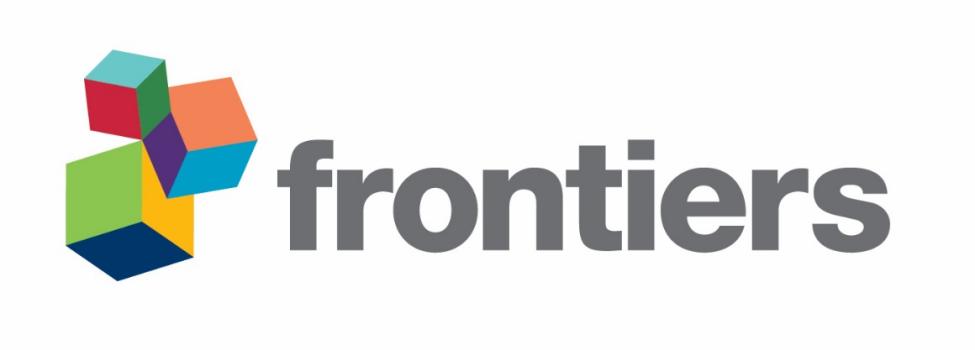
**
